# Identification of Three Clf-Sdr Subfamily Proteins in *Staphylococcus warneri*, and Comparative Genomics Analysis of a Locus Encoding CWA Proteins in *Staphylococcus* Species

**DOI:** 10.3389/fmicb.2021.691087

**Published:** 2021-07-29

**Authors:** Zhewei Sun, Xueya Zhang, Danying Zhou, Kexin Zhou, Qiaoling Li, Hailong Lin, Wei Lu, Hongmao Liu, Junwan Lu, Xi Lin, Kewei Li, Teng Xu, Mei Zhu, Qiyu Bao, Hailin Zhang

**Affiliations:** ^1^Key Laboratory of Medical Genetics of Zhejiang Province, Key Laboratory of Laboratory Medicine, Ministry of Education, School of Laboratory Medicine and Life Sciences, Wenzhou Medical University, Wenzhou, China; ^2^The Second Affiliated Hospital and Yuying Children’s Hospital, Wenzhou Medical University, Wenzhou, China; ^3^School of Laboratory Medicine and Life Sciences, Institute of Biomedical Informatics, Wenzhou Medical University, Wenzhou, China; ^4^Institute of Translational Medicine, Baotou Central Hospital, Baotou, China; ^5^Department of Clinical Laboratory, Zhejiang Hospital, Hangzhou, China

**Keywords:** cell wall anchored proteins, MSCRAMM, SdrJ/K/L, *Staphylococcus warneri*, comparative genomics, *sdr* loci

## Abstract

Coagulase-negative *Staphylococcus warneri* is an opportunistic pathogen that is capable of causing several infections, especially in patients with indwelling medical devices. Here, we determined the complete genome sequence of a clinical *S. warneri* strain isolated from the blood culture of a 1-year-old nursling patient with acute upper respiratory infection. Genome-wide phylogenetic analysis confirmed the phylogenetic relationships between *S. warneri* and other *Staphylococcus* species. Using comparative genomics, we identified three cell wall-anchored (CWA) proteins at the same locus (*sdr*), named SdrJ, SdrK, and SdrL, on the chromosome sequences of different *S. warneri* strains. Structural predictions showed that SdrJ/K/L have structural features characteristic of Sdr proteins but exceptionally contained an unusual N-terminal repeat region. However, the C-terminal repetitive (R) region of SdrJ contains a significantly larger proportion of alanine (142/338, 42.01%) than the previously reported SdrI (37.00%). Investigation of the genetic organization revealed that the *sdrJ/K/L* genes were always followed by one or two glycosyltransferase genes, *gtfA* and *gtfB* and were present in an ∼56 kb region bordered by a pair of 8 bp identical direct repeats, named Sw-Sdr. This region was further found to be located on a 160-kb region subtended by a pair of 160-bp direct repeats along with other virulence genes and resistance genes. Sw-Sdr contained a putative integrase that was probably a remnant of a functional integrase. Evidence suggests that Sw-Sdr is improbably an efficient pathogenicity island. A large-scale investigation of *Staphylococcus* genomes showed that *sdr* loci were a potential hotspot of insertion sequences (ISs), which could lead to intraspecific diversity at these loci. Our work expanded the repository of *Staphylococcus* Sdr proteins, and for the first time, we established the connection between *sdr* loci and phylogenetic relationships and compared the *sdr* loci in different *Staphylococcus* species, which provided large insights into the genetic environment of CWA genes in *Staphylococcus*.

## Importance

*Staphylococcus warneri* can occasionally cause infection in patients with a compromised immune system. The emerging of multidrug-resistant (MDR) strains among *Staphylococcus*, *S*. *warneri* and other coagulase-negative *Staphylococcus* species has attracted the attention of scientists. *Staphylococcus* as gram-positive bacteria can express variety of cell wall-anchored (CWA) proteins that are considered as major virulence factors on their surface. Unfortunately, the genetic environment of these important virulence genes has not been systematically studied. In this study, we report three different *sdr* genes, encoding cell wall anchored proteins that belong to Clf-Sdr Subfamily, which locate at the same locus (*sdr*) of the chromosome sequences of different *S. warneri* strains. Comparison of the CWA genes at *sdr* loci in different *Staphylococcus* strains revealed both interspecific and intraspecific diversities at this locus. Importantly, we established the connection between the *sdr* locus and phylogenetic relationship and proposed that the *sdr* locus might be the potential hotspot of recombination, which would lead to intraspecific diversity at this locus. Our research provides insight into genetic environment of CWA genes in *Staphylococcus*, which fills in the gap of this filed.

## Introduction

Staphylococci continue to be the leading cause of many human infections. Along with *Staphylococcus aureus* and other coagulase-positive staphylococcal (CoPS) strains, whose pathogenicity and mechanisms of virulence are well elucidated, the coagulase negative species has recently garnered more attention. Albeit less virulent than coagulase-positive isolates (they express a restricted number of virulence factors), nowadays they are the most prevalent microorganisms within the genus, particularly in hospital settings, where iatrogenic procedures have improved patient care, but have also promoted body invasion by such pathogens.

*Staphylococcus warneri*, along with *Staphylococcus epidermidis*, is one of the predominant coagulase-negative staphylococcal (CoNS) bacteria inhabiting the skin in healthy humans (Nagase et al., 2002). In comparison with *S. aureus* and other CoPS strains that can cause a range of illnesses, from minor skin infections to life-threatening diseases, *S. warneri* prefers to cause infections if the patients have underlying conditions such as indwelling foreign bodies and/or immunosuppression. As a common saprophyte of human epithelia, *S. warneri* is also frequently isolated from saliva, dental plaque, and nasal swabs, where it represents the third most prevalent CoNS species after *S. epidermidis* and *S. hominis* (Ohara-Nemoto et al., 2008). Over the past three decades, with the progressive refinement of identification techniques, *S*. *warneri* has been reported as a new pathogenic species frequently isolated from bacteremia, sepsis with multiple abscesses, orthopedic infections, vertebral osteomyelitis, and ventricular shunt infections of immunocompromised patients (Bryan et al., 1987; Kamath et al., 1992; Campoccia et al., 2010; Martínez-Lage et al., 2010). In addition, the overuse of antibiotics has led to a rapid increase in the occurrence of multiresistant strains of many bacteria, including *S. warneri*. Currently, the importance of *S*. *warneri* as a modern-day pathogen is growing, as it has established itself as a successful nosocomial pathogen.

Pathogenic gram-positive cocci express a plethora of virulence factors, including cell wall-anchored (CWA) proteins involved in microbial attachment to host tissues, which are considered the first critical step in the establishment of most bacterial infections (Foster and Höök, 1998). *S. aureus* can express up to 24 different CWA proteins, whereas CoNS species such as *S. epidermidis* and *S. lugdunensis* express a smaller number of CWA proteins (Bowden et al., 2005; Heilbronner et al., 2011). Timothy et al., proposed to classify *Staphylococcus* CWA proteins into four groups, namely, microbial surface component recognizing adhesive matrix molecule (MSCRAMM) family, near-iron transporter (NEAT) motif family, three-helical bundle family and G5–E repeat family, on the basis of the presence of motifs that have been defined by structure–function analysis (Foster et al., 2014). However, to the best of our knowledge, there are still numerous CWA proteins in *Staphylococcus* that cannot be assigned to any groups. The most prevalent group in *Staphylococcus* is the MSCRAMM family, which is defined by tandemly linked IgG-like folded domains (N2N3) that can engage in ligand binding by the dock, lock and latch (DLL) mechanism (Ponnuraj et al., 2003; Foster et al., 2014; Foster, 2019). The Clf-Sdr protein, with a repeat (R) region composed of serine-aspartate dipeptide repeats at the C-terminus, defines a subfamily of MSCRAMMs. The Clf-Sdr subfamily in *S. aureus* includes clumping factor A/B (ClfA/B) and SdrC/D/E (Foster et al., 2014), while in *S. epidermidis* it includes SdrG/F (Bowden et al., 2005). The N2N3 domains of these Sdr proteins share low sequence identities, which allow them to have different functions and target different ligands or the same ligands at different sites (Foster and Höök, 1998; Wang et al., 2013). For example, ClfA and SdrG have been shown to only bind to fibrinogen molecules at different sites, while ClfB is reported to adhere to multiple peptides carrying the glycine-serine-rich (GSR) motif, such as fibrinogen α (Fgα), cytokeratin 10 (CK10) peptides and dermokines (Ganesh et al., 2011; Xiang et al., 2012). Unfortunately, the MSCRAMMs in CoNS, except for *S. epidermidis*, draw less attention to researchers; for example, MSCRAMMs from *S. warneri* have not been characterized yet.

Cell wall-anchored proteins can be encoded on mobile genetic elements (MGEs), such as biofilm-associated protein (Bap) (Ubeda et al., 2003), *S. epidermidis* surface protein J (SesJ) (Arora et al., 2020) and *S. saprophyticus* surface protein F (SssF) (King et al., 2012). The presence of virulence factors in MGEs can influence the virulence potential of a specific strain. Therefore, it is critical to study the genetic organization of virulence factors in emerging pathogenic bacteria to understand the mechanisms employed by these bacteria to cause infections. Sdr proteins, as an interesting subfamily of MSCRAMMs, have attracted much attention from researchers, but most studies have focused on their interaction with host proteins. Based on previous studies, we found that some of these Sdr proteins were obviously a part of accessory genes, such as SdrD/E from *S. aureus* (Liu et al., 2015). However, what leads to such a situation remains elusive.

Here, we preliminarily characterized three different Sdr proteins at the same locus in *S. warneri* strains and performed a comprehensive comparative genomics analysis of the locus in different *Staphylococcus* species.

## Materials and Methods

### Bacterial Strains, Genome Sequencing, and Genome Assembly

*Staphylococcus warneri* WS479 was isolated from the blood culture of a 1-year-old nursling patient with acute upper respiratory infection in an affiliated hospital of Wenzhou Medical University, Zhejiang, China. The strain was identified by a Vitek-60 microorganism autoanalysis system (BioMerieux Corporate, Craponne, France).

The AxyPrep Bacterial Genomic DNA Miniprep Kit (Axygen Scientific, Union City, CA, United States) was used to extract the genomic DNA of *S. warneri* WS479. Library preparation, MinION sequencing and Illumina sequencing were performed at Shanghai Sunny Biotechnology Co., Ltd. The MinION long reads were initially assembled by Canu v1.8 (Koren et al., 2017), and then two FASTQ sequence files generated using the Illumina HiSeq 2500 platform were mapped onto the primary assembly to control assembly quality and to correct possible misidentified bases by using Bwa (Li and Durbin, 2009).

The genomes used to determine phylogenetic relationships and perform comparative genomics analysis were downloaded from the NCBI genome database. For those genomes used to investigate the *sdr* locus, the complete genome sequences were included preferentially, and the draft genome sequences that incompletely harbored the *sdr* locus were excluded. All the genomes used in this study were confirmed by calculating average nucleotide identity (ANI) (Supplementary Figure 5) using FastANI (Jain et al., 2018).

### Antimicrobial Susceptibility Testing and Cloning Experiments

The minimum inhibitory concentrations (MICs) were determined using the agar dilution method following the guidelines of the Clinical and Laboratory Standards Institute (CLSI, 2019). Susceptibility patterns were interpreted according to the CLSI breakpoint criteria for *Staphylococcus*. MIC was defined as the lowest concentration producing no visible bacterial growth. *Enterococcus faecalis* ATCC 29212 and *Escherichia coli* ATCC 25922 were used as reference strains for quality control. The resistance gene sequences (*aadD2* and *blaZ*) along with their promoter regions were PCR-amplified using the primers 5′-GCTCTAGAGCTTTCTATTATTGCAATGTGGAATTG-3′ and 5′-CGGGATCCCGTCAAAATGGTATGCGTTTTGACACA-3′ for *aadD2* and 5′-CGGGATCCCGATTTAGCCATTTTGACA CCTTCTTT-3′ and 5′-CCAAGCTTGGTTAAAATTCCTTCAT TACACTCTTGGCG-3′ for *blaZ*, with each having a pair of flanking restriction endonuclease adapters (*Xba*I and *Bam*HI for *aadD2* and *Bam*HI and *Hin*dIII for *blaZ*). The PCR products of *aadD2* and *blaZ* were then eluted from agarose gel, digested with the corresponding restriction endonucleases, and ligated into the pAM401 and pUCP24 vectors, respectively. The recombinant plasmid (pAM401-*aadD2*) was transformed into *E. faecalis* JH2-2 by the electroporation method, and the transformants were grown on brain heart infusion agar plates supplemented with chloramphenicol (16 μg/mL). The recombinant plasmid (pUCP24-*blaZ*) was transformed into *E. coli* DH5α cells via the calcium chloride method, and the transformants were grown on Luria-Bertani agar plates supplemented with gentamicin (40 μg/mL). The recombinant plasmids were verified by both restriction endonuclease digestion and Sanger sequencing (Shanghai Sunny Biotechnology Co., Ltd., Shanghai, China).

### Construction of a Phylogenetic Tree

To determine the phylogenetic relationship between *S. warneri* and other *Staphylococcus* species based on genomic data, we extracted and concatenated whole genome-wide single-copy orthologous protein sequences that were detected by OrthoFinder version 2.3.8 (Emms and Kelly, 2019) from 74 completely sequenced *Staphylococcus* genomes and a *Bacillus subtilis* genome (*Bacillus subtilis* 168, served as an outgroup). Multi-FASTA alignment was performed using MAFFT v7.407 (Katoh and Standley, 2013). Then, Gblocks (Castresana, 2000) was used to select conserved blocks of the resulting alignment. RAxML version 8.2.12 (Stamatakis, 2014) was used to infer the phylogeny by the maximum likelihood algorithm (ML) under the substitution matrix WAG which was chosen by ProtTest version 3.4 (Darriba et al., 2011).

### Sequence Analysis and Molecular Modeling

Identification of domains and other sequence features that occurred within proteins was carried out using InterProScan (Jones et al., 2014), SignalP^1^ and TMHMM^2^. The repeat domains were identified using T-REKS^3^. Furthermore, several positively charged residues and LPXTG motifs at the C-terminus of CWA proteins were checked and confirmed manually. Comparison of the identities among proteins was performed using BLASTN and BLASTP (Altschul et al., 1990). Multiple sequence alignment was carried out using ClustalW^4^. The homology models of the N2N3 domains of SdrJ and SdrL were generated using the SWISS-MODEL repository using the crystal structure of the N2N3 domains of ClfB and SdrC (PDB IDs: 3AU0 and 6LXH) as the template, respectively. The homology model of the N2N3 domains of SdrK was ultimately established using MODELER (Webb and Sali, 2014) based on the crystal structures of SdrE and SdrG. Subsequently, the quality of the final models was assessed by a Ramachandran plot using the RAMPAGE server and quality model assessment with the protein structure analysis (ProSA) server (Wiederstein and Sippl, 2007; Kaufmann et al., 2011).

### Protein Purification and Fluorescence Experiments

The DNA sequences of rSdrL178-748 (*S. warneri* WB224) and rSdrD54-680 (*S. aureus* TCH60) were synthesized with a pair of flanking restriction endonuclease adapters (*Nde*I for the forward primer and *Xho*I for the reverse primer) at Shanghai Tsingke Biotechnology Co., Ltd. Using pCold II as a cloning vector and *E. coli* BL21 as the recipient, transformants (pCold II-rSdr/BL21) were selected on LB agar plates containing 100 μg/mL ampicillin. The overnight culture of the recombinant strain (pCold II-rSdr/BL21) was diluted 100-fold in 100 ml of LB medium, incubated for 2–3 h at 37°C with orbital shaking at 250 rpm until the OD_600_ reached 0.6, and incubated with 1 mM isopropyl-β-d-thiogalactopyranoside (IPTG) (Sigma Chemicals Co., St. Louis, MO, United States) for 20 h at 16°C (Choi and Geletu, 2018). The recombinant protein was purified by affinity chromatography using BeyoGold His-tag Purification Resin (Beyotime, Shanghai, China) according to the manufacturer’s instructions. The histidine tag was removed by Enterokinase (GenScript, Nanjing, China) for 16 h at 16°C.

Bis-ANS (4,4′-dianilino-1,1-binaphthyl-5,5′-disulfonic acid, dipotassium salt) emission spectra were measured using a fluorescence spectrophotometer (F-7000). rSdrL178-748/rSdrD54-680 (3 μM monomer) in 50 mM Tris–buffer and 10 mM NaCl (pH 7.5), was incubated for 1 h at 25°C in absence or presence of 1 mM bivalent CaCl_2_ (Ca^2+^-bound) were saturated with 10 μM bis-ANS and their emission spectra were recorded from 430 to 600 nm using an excitation wavelength of 390 nm.

### Gene Predictions, Genomic Islands Predictions, and Functional Annotations

Genes were predicted and preliminarily annotated by using Prokka v1.14.0 (Seemann, 2014). Prediction of genomic islands (GIs) was carried out by combining a host of tools, such as Islandviewer 4 (Bertelli et al., 2017), PredictBias (Pundhir et al., 2008) and Alien Hunter (Vernikos and Parkhill, 2006). Direct repeats were identified using the inbuilt Find Repeat and Emboss tool fuzznuc in Geneious software. Easyfig (v2.2.3) software was used to generate the figures showing sequence comparisons and nucleotide identities of the *sdr* locus-containing region (Sullivan et al., 2011). Functional annotations were performed by querying different databases, such as the NCBI non-redundant (NCBI Resource Coordinators, 2018) and Swiss-Prot (UniProt Consortium, 2015) databases, using DIAMOND (Buchfink et al., 2015) with an evaluation threshold of 1e-5. Annotation of the resistance genes was performed using ResFinder (Zankari et al., 2012) and Resistance Gene Identifier (RGI) software version 4.0.3 of the Comprehensive Antibiotic Resistance Database (McArthur et al., 2013). Identification of virulence genes was carried out using BLASTP to search the protein sequences against the Virulence Factor Database (VFDB)^5^. Other bioinformatics tools were written using Python^6^.

### Data Availability

The complete chromosome and two plasmid sequences (pWS-31 and pWS-25) of *S. warneri* WS479 have been submitted to DDBJ/EMBL/GenBank under accession numbers, CP061041.1, CP061042.1, and CP061043.1, respectively.

## Results and Discussion

### General Features of the *S. warneri* WS479 Genome

The whole genome of *S. warneri* WS479 contained one single circular chromosome and two plasmids designated pWS-31 and pWS-25 (Supplementary Table 1). The chromosome is approximately 2.51 Mb in length (Supplementary Figure 1) and contains 2,491 ORFs, 5 rRNA-encoding gene (rDNA) clusters and 62 tRNA with an average GC content of 32.87%. The plasmids pWS-31 and pWS-25 have circularly closed DNA sequences of 31,000 bp and 25,160 bp in length with average GC contents of 29.86 and 30.46%, respectively.

The *in vitro* susceptibility testing of *S. warneri* WS479 exhibited resistance to a number of antibiotics (Supplementary Table 2), including erythromycin and clarithromycin (macrolides) and amikacin and azithromycin (aminoglycosides), according to CLSI breakpoint criteria for *Staphylococcus* (Supplementary Table 2). Moreover, the minimum inhibitory concentration (MIC) of roxithromycin against *S. warneri* WS479 was 256 μg/mL, which was significantly higher than the resistance breakpoint for erythromycin (>8 μg/mL), though no interpretation criteria for the antimicrobial was available. Moreover, three antibiotic resistance genes (*blaZ*, *aadD2* and *msr*A) involved in drug resistance against three antibiotics (β-lactams, aminoglycosides and macrolides, respectively) were predicated on the plasmid pWS-31, and two of them (*blaZ* and *aadD2*) were verified to be functional through cloning experiments (Supplementary Table 2).

### Phylogenetic Relationship Between *S. warneri* and Other *Staphylococcus* Species

**FIGURE 1:**
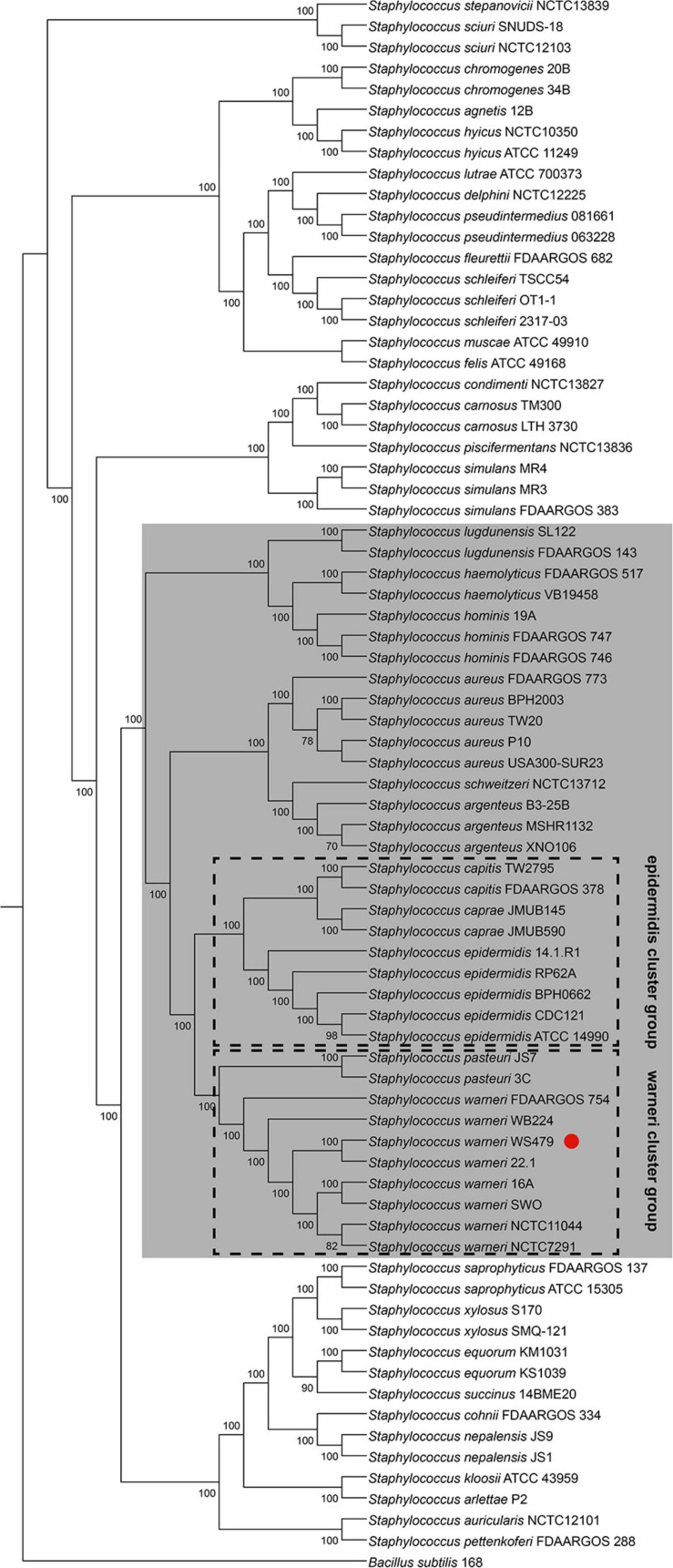
Phylogenetic tree for 74 complete genome sequences composed of 38 *Staphylococcus* species. The interior values are the bootstrap probabilities after 1,000 replicates. The species belonging to group SD are shaded in gray. *S*. *warneri* WS479 is indicated with a red dot.

Lamers et al. (2012) established a new classification using 16S rRNA, *tuf*, *rpoB* and *dnaJ* genes to classify the *Staphylococcus* species into 15 cluster groups. *S. warneri* together with *S. pasteuri* were defined as the “warneri cluster group.” To confirm the phylogenetic relationship between *S. warneri* and other *Staphylococcus* species, we constructed a maximum likelihood phylogenetic tree based on concatenating deduced amino acid sequences of 622 single-copy ortholog sequences from 74 completely sequenced *Staphylococcus* genomes (including *S. warneri* WS479) and a *Bacillus subtilis* genome (*Bacillus subtilis* 168, served as an outgroup) available in NCBI. The results confirmed that *S. warneri* together with *S. pasteuri* fell into a single clade with perfect supporting bootstrap values (100%) (Figure 1). Furthermore, the “warneri cluster group” was clustered closest to the “epidermidis cluster group,” which was composed of *S. epidermidis*, *S. capitis*, and *S. caprae*, indicating similar biological traits among these species.

### SdrJ/K/L Are Novel Clf-Sdr Subfamily Proteins

**FIGURE 2:**
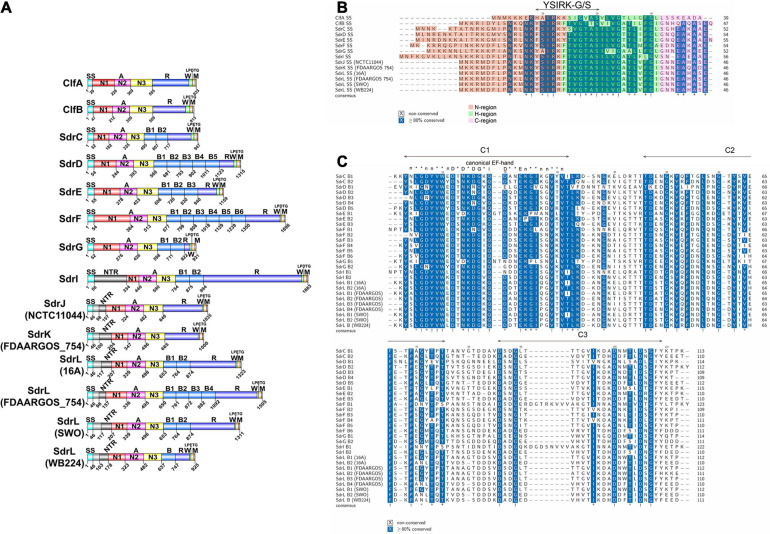
Comparison of the SdrJ/K/L proteins (only those with complete genome sequences and complete *sdr* genes are included). **(A)** Complete schematic representation of the *S. warneri* SdrJ/K/L proteins and other known Sdr subfamily proteins. The signal sequence is shown in light blue, NTRs in dark gray, N1 domain in red, N2 domain in pink, N3 domain in yellow, B-repeats in light blue, R region in dark blue, and cell wall-spanning region (W) in green and membrane anchor (M) in orange. The LPXTG motif is indicated directly. Strain names are shown in brackets. The accession number of SdrJ from *S. warneri* NCTC11044 is VED76443.1; SdrL from *S. warneri* 16A is QDW97251.1; SdrL and SdrK from *S. warneri* FDAARGOS_754 are QKI08103.1 and QKI08104.1, respectively; SdrL from *S. warneri* SWO is WP_122135109.1; SdrL from *S. warneri* WB224 is QJX56829.1; the accession numbers of ClfA/B and SdrC/D/E/F/G/I are BAF67028.1, BAF68801.1, BAF66795.1, BAF66796.1, BAF66797.1, AIR82723.1, AAF72510.1, and AF402316.1, respectively. **(B)** Multiple sequence alignments of the signal peptides in Clf/Sdr proteins. N, H, C regions are shadowed in red, green and pink, respectively. **(C)** Multiple sequence alignments of the B-repeats in Sdr proteins. Each B-repeat contains three conserved stretches, namely, C1, C2, and C3, which are framed in green boxes. The consensus sequence for a canonical EF-hand is taken from that of Kretsinger (1987). “*” means ≥80% conserved and “!” means 100% conserved.

When searching for the putative CWA proteins in *S. warneri* genomes, we discovered three genes encoding proteins sharing similar structures with the Clf-Sdr family, which we have called SdrJ/K/L, according to the established nomenclature. The deduced full-length SdrJ, SdrK and SdrL proteins in strains NCTC11044, FDAARGOS 754 and 16A are 1,035, 1,000, and 1,323 amino acids (aa) (Figure 2A) with predicted mature molecular masses of 100.56, 97.93, and 133.32 kDa, respectively. SdrJ/K/L are multidomain proteins that contain, starting from the N-terminus, a 46-amino acid long signal sequence, N-terminal repeats (NTRs), an A-region, 0–4 B-repeats, an SD (AD) repetitive (R) region and a typical cell wall anchoring sequence, such as an LPXTG motif, a hydrophobic membrane spanning region and a short cytoplasmic positively charged tail (Figure 2A). The A-region in these three proteins shares 35.90 to 45.36% sequence identity (Supplementary Table 3), suggesting that they have different functions. Surface proteins contain an N-terminal signal peptide that promotes their translocation across the bacterial membrane through the Sec pathway (Dramsi et al., 2008). The signal peptides of Sdr proteins are comprised a string of 17–18 hydrophobic aa (H-region), a variable-length N-region and an 8–10 aa C-region (Figure 2B). Multiple sequence alignments revealed that SdrJ/K/L like other reported Sdr proteins had a YSIRKXXXGXXS-like motif (Figure 2B). Deletion of this motif decreased the efficiency of signal peptidase, resulting in accumulation of precursors in the membrane and thus impacting on the secretion of CWA proteins (Dramsi et al., 2008).

**FIGURE 3:**
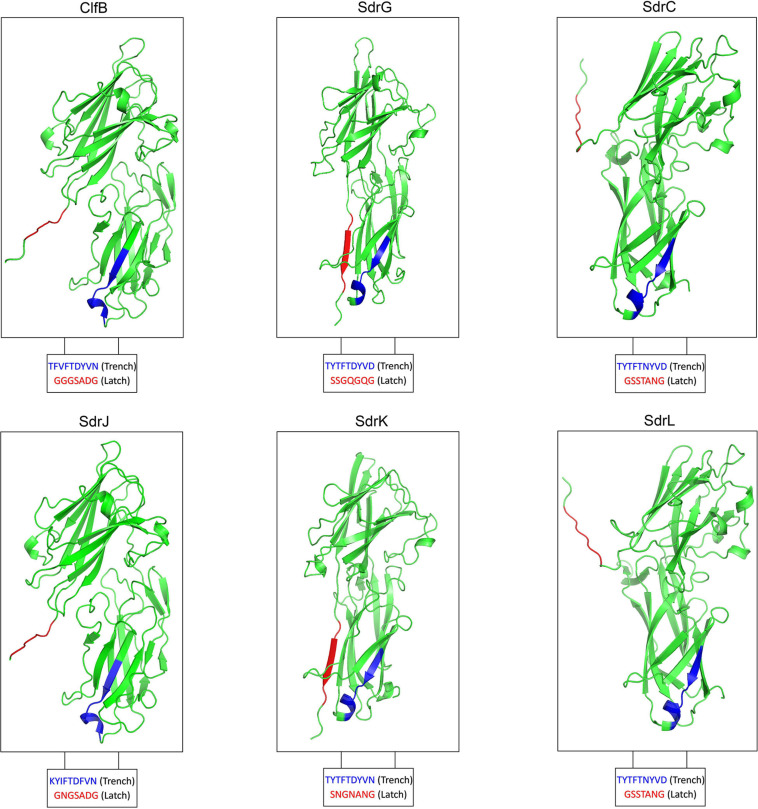
Modeled 3D structure of the N2N3 domains of SdrJ/K/L compared to the crystal structure of the N2N3 domains of ClfB, SdrG, and SdrC, respectively.

The most prevalent CWA proteins are MSCRAMMs, which are defined by tandemly linked IgG-like folded domains that bind to their ligands through the “dock, lock, and latch” (DLL) mechanism. This binding mechanism involves characteristic structural features in the A-region, including two adjacent IgG-like folded domains with a conserved TYTFTDYVD-like motif presenting at the “back” of the latching trench in the first domain in the tandem and a latch sequence at the C-terminal extension of the second domain (Ponnuraj et al., 2003). A latch sequence is not a conserved sequence of amino acids but is formed by alternating small residues (Ponnuraj et al., 2003). Domain and tertiary structure prediction revealed that residues 324–649 of SdrJ, 347–665 of SdrK and 339–653 of SdrL adopt two IgG-like folds (N2N3) (Figures 2A, 3). They all furthermore contained a TYTFTDYVD-like motif at the expected positions for a latching trench in the first domain of the predicted IgG-like tandem and a putative latch sequence in the extension of the second IgG-like folded domain (Figure 3). The presence of a latch sequence and a conserved TYTFTDYVD-like motif in the N2N3 subdomain indicated that they could bind a ligand peptide by the DLL mechanism. Overall, the A-regions of these three novel Sdr proteins contain the characteristic IgG-like folded tandem of a MSCRAMM.

The A-region of SdrJ shared the highest sequence identity (48.04%) with that of ClfB. In addition, the B-repeat, a Ca^2+^-binding domain, was absent in both ClfB and SdrJ, which have very similar structures (Figure 2A). Significantly, however, the R region of SdrJ contained a larger proportion of alanine (42.01%), even than the previously reported SdrI (37.00%) (Sakinc et al., 2006). The R regions of Clf-Sdr proteins are hypothesized to extend the A-region away from the cell surface to prevent obstruction of ligand binding by the cell wall (Hartford et al., 1997; Lizcano et al., 2012); however, whether a rich of alanine acids in the R region of SdrJ and SdrI would influence ligand binding is still unclear.

The A-region of SdrK was similar to that of SdrG at the amino acid level (53.77%), suggesting that it may also have fibrinogen-binding activity (Davis et al., 2001; Sellman et al., 2008). B-repeats not only help project the A-region on the cell surface but also engage in ligand binding. For example, SdrF binds type I collagen via its B-repeats in a temperature-dependent manner (Di Poto et al., 2015). Thus, the absence of B-repeats in SdrJ and SdrK might weaken their ligand binding activity.

**FIGURE 4:**
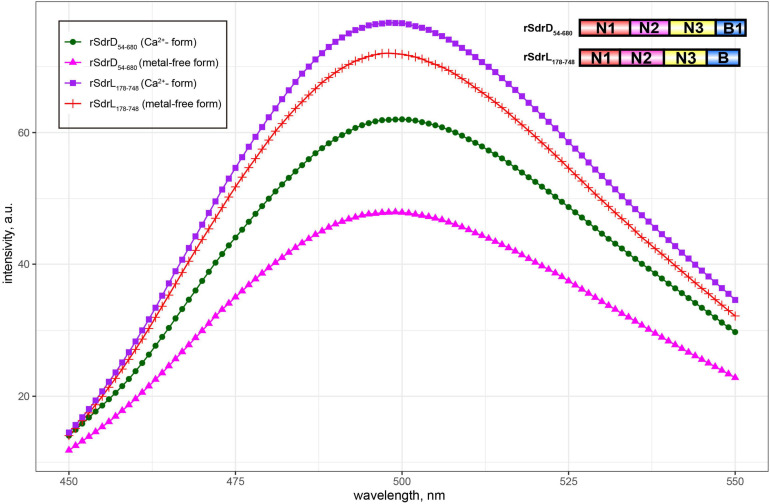
Fluorescence spectra of bis-ANS in the presence/absence of Ca^2+^-loaded (1 mM CaCl_2_) rSdrL_178__–__748_ and rSdrD_54__–__680_.

SdrL proteins are more prevalent than SdrJ and SdrK proteins in *S. warneri*. The A-region shared a significant sequence identity (73.50%) with that of SdrC, which was reported to involve β-neurexin binding as well as bacterial biofilm formation (Barbu et al., 2010, 2014). The B-repeats of *S. warneri* SdrL can vary from 1 to 4 copies. Sequence alignments from multiple B-repeats revealed that they also contained one conserved EF-hand motif (Figure 2C; Roman et al., 2016). To confirm the calcium binding ability of SdrL and investigate its change of hydrophobic surface in the presence of Ca^2+^, we purified rSdrL_178__–__748_ (A-region and one B-repeat) protein of *S. warneri* WB224 and rSdrD_54__–__680_ of *S. aureus* TCH60 (positive control). bis-ANS showed that compare with rSdrD_54__–__680_, rSdrL_178__–__748_ had less decrease in fluorescence (14.23 a.u. versus 4.88 a.u.) when in the presence of Ca^2+^ at 500 nm (Figure 4), which suggested that the B repeat of SdrL was functional. In summary, SdrL may be related to biofilm formation, and its predominance indicates its importance to the pathogenicity of *S. warneri*.

Staphylococcal MSCRAMMs do not usually contain an NTR domain, which is found in these three proteins between the signal sequence and the A-region (Arora et al., 2016). Unlike the NTR domains in SesJ and SdrI, the NTR domains found in the three *S. warneri* Sdr proteins were relatively short, and the tandem repeats were not identical (Table 1). As a result of the unusually short N1 region that connects the N2N3 tandem to the preceding NTR region, the A-region of these three proteins is smaller than that of their closest homologs. MSCRAMM family proteins bind to host proteins and can mediate bacterial adhesion, evade the immune response and participate in biofilm formation. Sequence analysis and modeling studies reveal that SdrJ/K/L harboring an unusual NTR domain are novel members of the *Staphylococcus* Clf-Sdr subfamily, which provides a point of penetration for further research on the pathogenicity of *S. warneri.* It is noteworthy that, *S. warneri* is the first CoNS possessing structural homologs (SdrJ/K) of Clf proteins that are free of B repeats. The interactions of SdrJ/K/L and their possible roles in *S. warneri* pathogenesis are currently being investigated.

**TABLE 1:** Summary of NTRs of the SdrJ/K/L proteins.

Protein (strain)	Sequence of NTR	Repeat number/Length of repeat sequence
SdrJ (NTCT11044)	EALTTAEEPKAEKTK EAPATAEEPKAEKTK EAPATAEEPKVDTTK EAPATAEKPKADTTK	4/15
SdrL (16A)	TEGAPKADTTKEESA- TEEAPKADTTKEAPA- TEEEPKADTTKEAPA- TAEEPKADTTKEAPT- TEEEPKADTTKEAPVT TEEQTPKTTTDQAPE-	6/15
SdrL (FDAARGOS 754)	EASTTEKEPKADTTK EASTTEEAPKADTTK EAPTTAEELKVETTK EEPATAEKPKADTTK	4/15
SdrL (SWO)	TEGAPKADTTKEESA- TEEAPKADTTKEAPA- TEEEPKADTTKEAPA- TAEEPKADTTKEAPT- TEEEPKADTTKEAPVT TEEQTPKTTTDQAPE-	6/15
SdrL (WB224)	——EETPKADTTKEAPTA- ——EEPKADTTKEKATTK EAPTTAEEPKADTTK——- EAPVTAEKPETDTTKE—— EA-TTEEAPKVDTTKE——	5/15
SdrK (FDAARGOS 754)	-STTEEAPKADTTKEE -STTEEAPKADTTKEE PATTEEAPKADTTKE- ESVTEEAPKADATKE- ESATEEAPKADATKE- ESATEEAPKADTTKE- ESTTEEAPKADTTKE-	7/15

### Genetic Organization of the Region Encoding SdrJ/K/L in *S. warneri*

**FIGURE 5:**
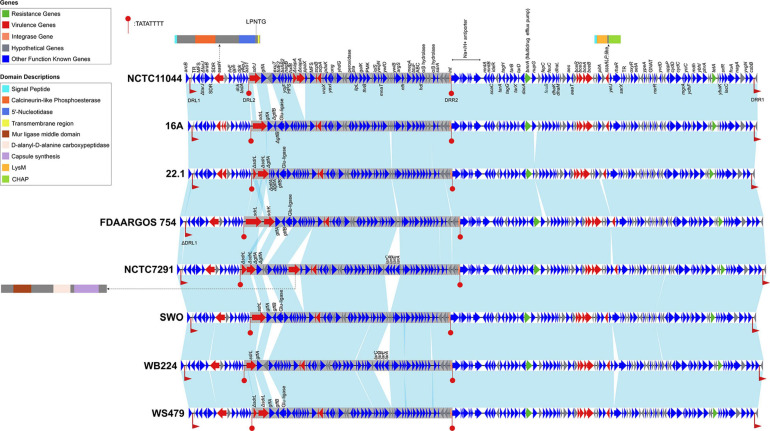
Genetic organization of the 160 kb region containing Sw-Sdr. Genes are shown as arrows and colored based on gene function classification (virulence genes in red and resistance genes in green). The light blue shading denotes regions of homology. The ∼ 56 kb Sw-Sdrs are in gray background. DRL1 and DRR1 indicate 160 bp direct repeats, and DRL2 and DRR2 indicate 8 bp prefect direct repeats. Domains of proteins encoded by three virulence genes (*sasH*, *capA*, and *ssaALP-*like genes) are shown in cartoons. Strain names are in boldface on the right side of each track.

Comparative genomic analysis revealed that SdrJ/K/L were encoded on the same locus (hereafter named the *sdr* locus), which was located between *azo1* (encoding FMN-dependent NADPH-azoreductase) and *folE2* (encoding GTP cyclohydrolase) on the chromosome of *S. warneri*. Bioinformatic prediction revealed that there were predicted genomic islands surrounding the locus encoding SdrJ/K/L. A search of direct repeat sequences resulted in finding an ∼160 kb segment of the *S. warneri* chromosome, including the *sdrJ/K/L* genes flanked by 160 bp direct repeats (DRL1 and DRR1) (Figure 5 and Supplementary Figure 2). Approximately 40 kb upstream of the segment was a cluster of tRNA genes. Moreover, within the 160 kb segment, an ∼56 kb subregion (hereafter named Sw-Sdr) was found to be bordered by 8 bp perfect direct repeats (Figure 5).

In total, we identified 9 or 10 putative virulence genes (depending on the strains) and two resistance genes in the 160 kb region (Figure 5). Notably, the *sdrJ/K/L* genes and their immediate surroundings within Sw-Sdr seemed to be poorly conserved. These *sdrJ/K/L* genes were always followed by one or two glycosyltransferases, *gtfA* and *gtfB* (Figure 5). Similar organization was also observed in other *Staphylococcus* species, such as downstream of the *sdrG* gene and *sdrC/D/E* genes in *S. epidermidis* and *S. aureus*, respectively (Hazenbos et al., 2013). These *gtf* genes were proposed to glycosylate the R regions of Sdr proteins, which can protect Sdr proteins against host proteolytic activity and yet generate major epitopes for the human anti-staphylococcal antibody response (Hazenbos et al., 2013). Thus, SdrJ/K/L might also subject to similar posttranslational modifications.

On the right side of Sw-Sdr was a putative tyrosine-type integrase that was 185 aa in length. A domain search revealed that the integrase only possessed a catalytic domain and had several key residues substituted at the RHRY tetrad (Supplementary Figure 3; Kwon et al., 1997); thus, it was an atypical integrase or might be a remnant of a functional integrase (Jo et al., 2016). Further BLAST analysis revealed that such atypical integrase seems to be frequently present in *Staphylococcus* genomes, but its detailed function remains unknown. Additionally, phylogenetic analysis revealed that these shortened integrases were phylogenetically distinct from known integrases of bacteriophages, integrons, integrative conjugative elements and other GIs (Supplementary Figure 4).

Overall, the genetic environment of *sdr* loci in *S. warneri* also harbored other virulence and resistance determinants; however, whether the region is a pathogenicity island remains unknown due to a lack of convincing evidence (this region has a similar GC content than the overall *S. warneri* genome (Supplementary Figure 1), is free of efficient mobility genes, and is free of genes from phages) (Hacker et al., 1997).

### Not Only MSCRAMM Genes but Also Other CWA Genes Can Be Found at the *sdr* Locus

Different *S. warneri* strains possessed different *sdr* genes at the *sdr* locus (between *azo1* and *folE2*), suggesting that these *sdr* genes were a part of accessory genes or might be present in an MGE. After a large-scale investigation of *Staphylococcus* genomes, we found that species harbored CWA genes and (or) *gtf* genes at the *sdr* locus fell into a single clade (Figure 1), namely, group SD, while the remaining species contained transcriptional regulator or metabolism -related genes instead of CWA genes and (or) *gtf* genes (data not shown).

**FIGURE 6:**
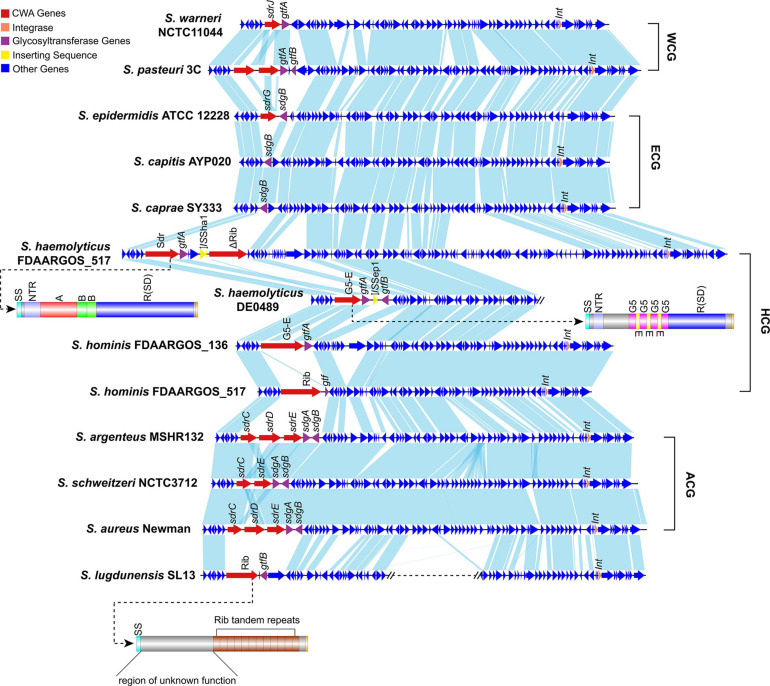
Comparison of Sw-Sdr (*S. warneri* NCTC11044) with related regions in other *Staphylococcus* species. Domains of the Sdr protein in *S. haemolyticus*, the G5-E family protein in *S. hominis* and the Rib domain containing protein in *S. lugdunensis* are shown. WCG, species belonging to the “*warneri* cluster group”; ECG, species belonging to the “*epidermidis* cluster group”; HCG, species belonging to the “*haemolyticus* cluster group”; ACG, species belonging to the “*aureus* cluster group”.

To better characterize Sw-Sdr, we compared Sw-Sdr (*S. warneri* NCTC11044) with other relative regions from different *Staphylococcus* species. The results showed that except for the region containing CWA genes and their immediate surroundings, the other regions were relatively conserved among the investigated *Staphylococcus* species. In *S. aureus* and *S. epidermidis*, the *sdr* locus harbored the well-known Sdr family proteins SdrC/D/E and SdrG, respectively (Figure 6). In some *Staphylococcus* species, such as *S. capitis* and *S. caprae*, the *sdr* locus was free of CWA genes but still possessed the *gtf* gene. In *S. haemolyticus*, CWA genes at the *sdr* locus could encode not only MSCRAMM proteins but also G5-E repeat family proteins and Rib domain-containing proteins (Figure 6 and Table 2). Rib domain-containing proteins could also be found at the *sdr* loci in *S. hominis* and *S. lugdunensis*. Such proteins feature a C-terminal of tandem repeated Rib domains that are thought to define protective epitopes and may play a role in generating phenotypic and genotypic variation in group B *Streptococcus* (Michel et al., 1992; Wästfelt et al., 1996). These facts suggest that the downstream integrase was independent of the diversity of CWA genes at the *sdr* locus because in *S. epidermidis*, the downstream integrase was absent, while in *S. lugdunensis*, the *sdr* locus and the integrase-containing region were not continuous. The integrase might be associated with the initial integration of this region in *Staphylococcus* but lost its function during evolution, thereby leading to the settling of this region.

**TABLE 2:** Statistics of CWA genes and ISs at *sdr* loci in 11 *Staphylococcus* species belong to group SD.

Species (Complete/Draft)	*S. aureus* (150/0)	*S. argenteus* (6/33)	*S. schweitzeri* (0/4)	*S. epidermidis* (45/39)	*S. caprae* (*n* = 5/0)/ *S. capitis* (4/22)	*S. haemolyticus* (*n* = 14/27)	*S. hominis* (12/11)	*S. lugdunensis* (25/5)	*S. warneri* (8/27)	*S. pasteuri* (2/5)
CWA proteins	SdrC (99.33%; *n* = 149) SdrD (92%; *n* = 138) SdrE (90%; *n* = 135)	SdrC-like (92.31%; *n* = 36) SdrD-like (79.49%; *n* = 31) SdrE-like (79.49%; *n* = 31)	SdrC-like (100.00%; *n* = 4) SdrD-like (25.00%; *n* = 1) SdrE-like (100.00%; *n* = 4)	SdrG (86.9%; *n* = 73)	NA	Rib domain containing (29.27%; *n* = 12) G5-E repeat family (31.71%; *n* = 13) Sdr family (56.10%; *n* = 23)	Rib domain containing (60.87%; *n* = 14) G5-E repeat family (39.13%; *n* = 9)	Rib domain containing (100.00%; *n* = 30)	SdrJ (8.57%; *n* = 3) SdrK (5.71%; *n* = 2) SdrL (88.57%; *n* = 31)	SdrL-like (85.71%; *n* = 6) SdrM (57.14%; *n* = 4)
CWA proteins positive rate	100.00%; *n* = 150	100.00%; *n* = 39	100%; *n* = 4	86.9%; *n* = 73	0.00%; *n* = 0	90.24%; *n* = 37	91.30%; *n* = 21	100.00%; *n* = 30	100%; *n* = 35	85.71%; *n* = 6
ISs	*IS*30 family (5.33%; *n* = 8) *IS*L3 family (1.33%; *n* = 2) *IS*256 family (2.00%%; *n* = 3) *IS*1182 family (1.33%; *n* = 2)	NA	NA	*IS*Sep3 (2.38%; *n* = 2)	NA	*IS*256 family (4.88%; *n* = 2) *IS*1182 family (39.02%; *n* = 16) *IS*L3 family (31.71%; *n* = 13)	*IS*1182 family (4.35%; *n* = 1)	NA	NA	NA
ISs positive rate	10.00%; *n* = 15	0.00%; *n* = 0	0.00%; *n* = 0	2.38%; *n* = 2	0.00%; *n* = 0	58.54%; *n* = 24	4.35%; *n* = 1	0.00%; *n* = 0	0.00%; *n* = 0	0.00%; *n* = 0

*For CWA genes in *S. haemolyticus*, *S. hominis* and *S. lugdunensis*, CWA genes are grouped based on presence key domains (N2N3 domains, B-repeats, G5-E domain, and Rib domain), and for other species, CWA genes are grouped based on sequence identities (Supplementary Figure 6).*

### Insertion Sequences at *sdr* Locus

We noticed that within a *Staphylococcus* species, although the CWA genes at this locus varied from strain to strain, the repository of these CWA genes was settled. To prove this, we investigated the genome sequences of the 11 *Staphylococcus* species belonging to group SD (Figure 1). All the investigated species showed a settled repository of CWA genes at this locus, such as *sdrC/D/E* in *S. aureus*, *sdrG* in *S. epidermidis*, and *sdrJ/K/L* in *S. warneri* and a gene encoding the Rib domain-containing protein in *S. lugdunensis* (Table 2). Moreover, species belonging to the same cluster group tended to possess similar CWA genes at the *sdr* locus; for example, the *sdrC/D/E* derivatives in *S. argenteus* and *S. schweitzeri* share high sequence identities (>80%) with *sdrC/D/E* genes in *S. aureus*.

We serendipitously observed that some *Staphylococcus* genomes occasionally integrated an insertion sequence (IS) at the *sdr* locus. More specifically, we noticed that 10.00% (15/150) of *S. aureus* strains possessed IS at this locus, probably due to a lack of complete genome sequences; only 2.38% (2/84) of *S. epidermidis* strains and 4.35% (1/23) of *S. hominis* strains were found to carry IS (Table 2). Complete genome sequence data are essential for the analysis of genes containing repeat regions, such as the *sdr* locus. Unfortunately, due to lack of genome resources, the IS element was not observed at the *sdr* locus in some *Staphylococcus* species. *S. haemolyticus* was a special case with more than half (58.54%; 24/41) of the investigated genomes possessing the IS element at the *sdr* locus; of note, 78.57% (11/14) of the completely sequenced strains possessed the IS element at the *sdr* locus, implying that this number would be higher if more complete genome sequences were included. IS element was reported to involve in the modulation of expression of virulence factors in many bacterial species including *Staphylococcus* (Ziebuhr et al., 1999; Rajeshwari and Sonti, 2000; Conlon et al., 2004; Simser et al., 2005; Perez et al., 2015). In our dataset, *S. lugdunensis* was the only species with a relatively large number of complete genomes that no IS element was identified at *sdr* loci. Further investigation revealed that the Rib domain-containing protein in *S. lugdunensis* was not similar with other Sdr or G5-E proteins that possessed R region. Thus, the repeat region of Sdr or G5-E proteins at *sdr* loci could act as a recombination hotspot for the integration of such mobile genetic elements. However, further experiments will be needed to confirm this hypothesis. Therefore, we thought that the intraspecific differences in CWA genes or truncation of CWA genes (such as *sdrL* in *S. warneri* NCTC7291 and *S. warneri* WS479) at this locus were caused by these mobile genetic elements.

However, it is still unclear whether the putative site-specific integrase downstream of the *sdr* locus is involved in the formation of different categories of CWA proteins in various *Staphylococcus* species. Since *sdr* loci variably contained CWA genes within a *Staphylococcus* species, it can be a potential locus used for typing strains with different pathogenicity.

## Conclusion

In this study, we determined the complete whole-genome sequence of *S. warneri* WS479, and confirmed that *blaZ* and *aadD2* on pWS-31 were functional. The phylogenetic relationship between *S. warneri* and other *Staphylococcus* species was confirmed by a genome-wide phylogenetic tree, and *S. warneri* together with *S. pasteuri* were in a single clade. Combining sequence analysis and modeling studies, we identified three CWA proteins, namely, SdrJ/K/L, on the chromosomes of *S. warneri*. These proteins have structural features characteristic of Sdr proteins but also contain an unusual N-terminal repeat region. Further investigation of the genetic organization of *sdrJ/K/L* revealed that they were actually located at the same locus (*sdr* locus) on the chromosome sequence. Although a putative integrase and direct repeats were observed, our analysis still suggested that the region containing Sdr proteins (Sw-Sdr) was unlikely to be an efficient pathogenicity island. Comparative genomic analysis showed that Sw-Sdr was a conserved region found in different *Staphylococcus* species, and the MSCRAMM gene was not the only CWA gene at *sdr* loci. A large-scale investigation of *Staphylococcus* genomes showed that *sdr* loci were a potential hotspot of insertion sequences (ISs), leading to intraspecies diversity at these loci.

## Data Availability Statement

The complete chromosome and two plasmid sequences (pWS-31 and pWS-25) of S. warneri WS479 have been submitted to DDBJ/EMBL/GenBank under accession numbers, CP061041.1, CP061042.1, and CP061043.1, respectively.

## Ethics Statement

This study was approved by The Ethics Committee of the Affiliated Hospital of Wenzhou Medical University (China) and informed consent was obtained from the patient.

## Author Contributions

ZS conceived the project and wrote the manuscript. DZ, XZ, and KZ performed the experiments. TX, QB, HZ, and MZ analyzed the results. All authors were involved in the design of the experiments and reviewed the manuscript.

## Conflict of Interest

The authors declare that the research was conducted in the absence of any commercial or financial relationships that could be construed as a potential conflict of interest.

## Publisher’s Note

All claims expressed in this article are solely those of the authors and do not necessarily represent those of their affiliated organizations, or those of the publisher, the editors and the reviewers. Any product that may be evaluated in this article, or claim that may be made by its manufacturer, is not guaranteed or endorsed by the publisher.

## Footnotes

1
http://www.cbs.dtu.dk/services/

2
www.cbs.dtu.dk/services/TMHMM-2.0/

3
https://bioinfo.crbm.cnrs.fr/index.php?route=tools&tool=3

4
https://embnet.vital-it.ch/software/ClustalW.html

5
http://www.mgc.ac.cn/VFs/

6
https://www.python.org/

## Supplementary Material

Supplementary Figure 1 Chromosomal comparison of *S. warneri* WS479 with 7 other *S. warneri* chromosome sequences. Strain names are shown in the legend. The innermost circle shows the scale in Mb.

Supplementary Figure 2 Multiple sequence alignments of the 160 bp DRL1s/DRR1s.

Supplementary Figure 3 Multiple sequence alignments of the putative integrases on Sw-Sdr in *S. warneri* or Sw-Sdr-related regions in other *Staphylococcus* species with Cre, XerD, and phage integrase. The RHRY tetrad is shaded in gray.

Supplementary Figure 4 The inferred phylogenetic relationship of the putative integerase on Sw-Sdr in *S. warneri* or Sw-Sdr related regions in other *Staphylococcus* species belong to group SD in relation to representative tyrosine recombinases of the shufflon-specific DNA recombinase (Rci), dusA-associated integrases (DAI), phage integrase (Phage), integrative and conjugative element (IntC), *Staphylococcus* pathogenicity island (SPI), *Staphylococcus* resistance island (SRI), integron integrase (IntI), site-specific recombinase (XerDC), phage P1-like recombinases (Cre) and genomic island integrase (IntG) families. Protein accession numbers of sequences used to generate this phylogenetic tree are in brackets. The interior values are the bootstrap probabilities after 1,000 replicates.

Supplementary Figure 5 Average nucleotide identity between each genome used to investigate the *sdr* locus.

Supplementary Figure 6 Sequence identity comparison of CWA proteins (identified based on the presence of key domains) encoded at *sdr* loci in *Staphylococcus* species belonging to group SD.

Supplementary Table 1 Genome features of *S. warneri* WS479.

Supplementary Table 2 MIC values of antibacterial drugs for all strains (μg/mL).

Supplementary Table 3 Sequence identity matrix table containing all the SDR subfamily proteins compared in terms of SS, A, B, R, W, and M regions.
