# Supplementary material for: Identification of Three Clf-Sdr Subfamily Proteins in Staphylococcus warneri, and Comparative Genomics Analysis of a Locus Encoding CWA Proteins in Staphylococcus Species
Source: Front Microbiol. 2021 Jul 29;12:691087. doi: 10.3389/fmicb.2021.691087 (PMC8360574; doi:10.3389/fmicb.2021.691087)
Supplement: Supplementary Figure 2 — Multiple sequence alignments of the 160 bp DRL1s/DRR1s. [file Image_2.PDF]

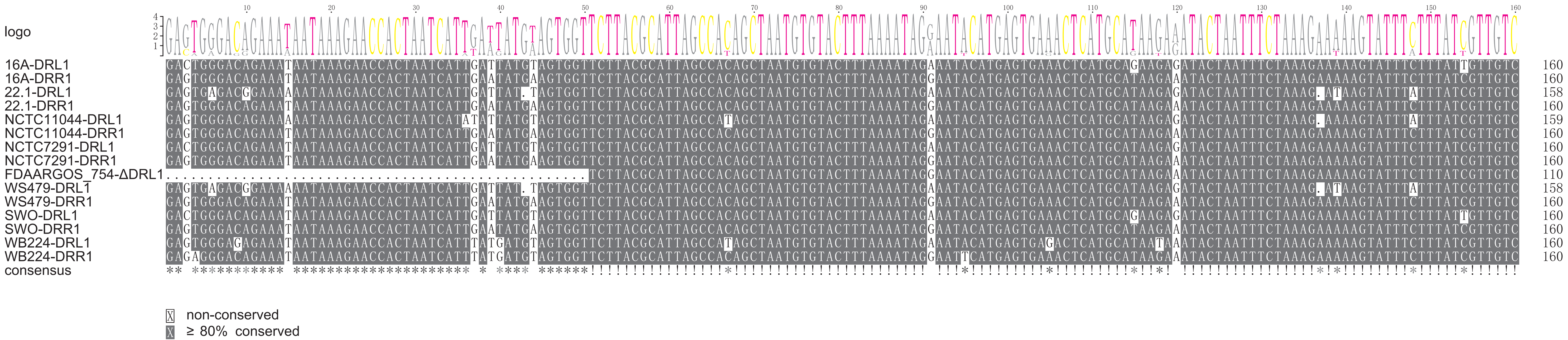
logo
16A-DRL1 160
16A-DRR1 160
22.1-DRL1 158
22.1-DRR1 160
NCTC11044-DRL1 159
NCTC11044-DRR1 160
NCTC7291-DRL1 160
NCTC7291-DRR1 160
FDAARGOS_754-ΔDRL1 110
WS479-DRL1 158
WS479-DRR1 160
SWO-DRL1 160
SWO-DRR1 160
WB224-DRL1 160
WB224-DRR1 160
consensus
non-conserved
≥ 80% conserved
