# Supplementary material for: Identification of Three Clf-Sdr Subfamily Proteins in Staphylococcus warneri, and Comparative Genomics Analysis of a Locus Encoding CWA Proteins in Staphylococcus Species
Source: Front Microbiol. 2021 Jul 29;12:691087. doi: 10.3389/fmicb.2021.691087 (PMC8360574; doi:10.3389/fmicb.2021.691087)
Supplement: Supplementary Figure 3 — Multiple sequence alignments of the putative integrases on Sw-Sdr in S. warneri or Sw-Sdr-related regions in other Staphylococcus species with Cre, XerD, and phage integrase. The RHRY tetrad is shaded in gray. [file Image_3.PDF]

```
E. gerundensis Cre   ............MTDSLPAPLPLHALSADADISARLAEFVRDKDAFSPNTWRQLLSVMRICFSWSQQNGRSFLPMSPDDLRDYLTHLQEIGRASSTISTHASLISMLHRNAGLVPPNTSPAVFRTMKKINRVAVIAGERTGQAVPFRLNDLMALDRCWVN.....   148
E. coil XerD         .........................MKQDLARIEQFLDALWLEKNLAENTLNAYRRDLSMMVEWLHHRGLTLATAQSDDLQALLAERLEGGYKATSSARLLSAVRRLFQYL..............YREKFREDDPSAHLASPKLPQRLPKDLSEAQVERLLQAPL   126
Haemophilus phage    MAVRKDTKNGKWLAEVYVNGNASRKWFLTKGDALRFYNQAKEQTTSAVDSVQVLESSDLPALSFYVQEWFDLHGKTLSDGKARLAKLKNLCSNLGDPPANEFNAKIFADYR..............KRRLDGEFSVNKNNPPKEATVNREHAYLRAVFN.......   144
S. warneri           ...........................................................................................MNQVEPIRRTEDIQKMYSVL..............RSRSQR..................................    26
S. pasteuri          ...........................................................................................MNQVEPIRNTEDIQKMYTIL..............RSRSQR..................................    26
S. capitis           ...........................................................................................MNQVDPIKYKEEIHQMYQVL..............QERSQR..................................    26
S. caprae            ...........................................................................................MNQVEPIKHKEEIYQMYKVL..............QERTQR..................................    26
S. haemolyticus      ...........................................................................................MNQVEPIRNKDDIKKMYDVL..............KKKSDR..................................    26
S. hominis           ...........................................................................................MNYVEPIKKKNDIKKMYHVL..............QMKSDR..................................    26
S. schweitzeri       ...........................................................................................MNKVEAIKKNEDIIKMYQVL..............KHKSER..................................    26
S. aureus            ...........................................................................................MNKVEAIKFNDDIVKMYEAL..............KIKSER..................................    26
S. lugdunensis       ...........................................................................................MNQVEPIRNKEDIKRMYQVL..............SMKSKR..................................    26

E. gerundensis Cre   ATRLQDLRNLAFLHIAYGTLLRVSELARLRVRDVTRAEDGRIILDVAWTKTIVQTG...GLIKALSALSTRRLEAWIAAAGLAREPDAFLFCRVHRCNKALLTEEAPLSTPAIEAIFSHAWQTIGPAEPARANKSRYRGWSGHSARVGAAQDMAKQGYAVAQIMQ   310
E. coil XerD         IDQPLELRDKAMLEVLYATGLRVSELVGLTMSDIS..LRQGVVRVIGKGNKER......LVPLGEEAVYWLETYLEHGRPWLLNGVS...............IDVLFPSQRAQQMTRQTFWHRIKHYAVLAGIDSEKLS..PHVLRHAFATHLLNHGADLRVVQM   266
Haemophilus phage    .......ELKSLRKWTTENPLDGVRLFKERETELAFLYERDIYRLLAECDNSRNPDLGLIVRICLATGARWSEAETLTQSQVMPYKITFTNTKSKKNRTVPISKELFDMLPKKRGRLFNDAYESFENAVLRAEIELPKGQLTHVLRHTFASHFMMNGGNILVLKE   302
S. warneri           ........DYLLFKFAIHTGIKLSELLNMSVQRLK.SIESGNIKTTWIDDGEL......TIQILLPPDLRNELQDYIDYYEIKE..................NDLVFQSIRTGKGLSRQQAYRIINKAAEE.LDIPHIG..LTTLRKTFAYHAYQSGISISIIQK   155
S. pasteuri          ........DYLLFKFAIHTGVKLCELLNMKVHRLK.SVESGNIKSTWIDNVDP......SIQILLPPDLRNELQDYINHYQIQE..................DKLIFQSLRTGKGLSRQQAYRIIHKAAEE.SNIPHVG..LTTLRKTFAYHAYQSGISISIIQK   155
S. capitis           ........DYLFFKFAIHTGIKLTDLLNMKVKYLK.TSEIGNIKTSWIIDDEP......TIKIKLPSELRNELKLYIEDCKLED..................EELIFQSTRTHQCLSRQQAYRIINRAAEQ.LGMKHIG..LTTLRKTFAYHAYQAGISISIIQK   155
S. caprae            ........DYLFFKFAIHTGVKLTELLNMKVKHLK.MTETGNIKTAWIVDYEP......SIKIMLPTELQLEIKSYIEEHHLKD..................EDLVFRSMRTQQCLSRQQAYRIIHQAAAR.LGMRHIG..LTTLRKTFAYHAYQSGISISIIQK   155
S. haemolyticus      ........DYLLFKLAIHTGIRLTDLLNLKVKDVK.MIDQDEIKSSWIESCAP......AIKILLPNDLREEVRRFIEYNKLMD..................DHLLFQSIRTHKELSRQQAYRIIHHAAEE.LGLCHIG..LTTLRKTFAYHAYQSGISIAIIQK   155
S. hominis           ........DYLLFKLAIHTGMRLTDLLHLKVEDIK.NKENKEIKTAWIQQSAP......FIKIMIPSDLRNEIDTYIYENQLKD..................DHLLFQSLRTHKELSRQQAYRIIHHAAKE.LGLLHIG..LTTLRKTFAYHAYKSGISIAIIQK   155
S. schweitzeri       ........DYLFFKLAIHSGLKMSDLLALTVEQVNVLIDNCRLSELCKVHYHS......LIKIKLPESLSKELLHYIKQKNLSN..................EDFLFQSLRTNQVLSRQQAYRIIHKAAKE.ANIEHVG..LTTLRKTFAFHAYQKGIPIVIIQK   156
S. aureus            ........DYLFFKLAIHSGLKVSELLTITVSQVKRLIEKCTLSEMCKAHFHS......LIKIRLPETLSKELLQYIEDRSLSN..................EDVLFQSLRTNQVLSRQQAYRIIHQASIE.AGIDNVG..LTTLRKTFAYHAYQKGIPIPVIQK   156
S. lugdunensis       ........DYLFFKLAIHTGIRLTDLLKLEVKDIK.NLQRGNIKTAWIENTVP......SIKIKLPETLRQELTDYIDDYQLAN..................QQLLFQSIRTHQALSRQQAYRIIHQAAEE.LGILHIG..LTTLRKTFAYHAYESGISISIIQK   155

E. gerundensis Cre   EGTWKKPETLMRYIRNIDAHQGAMVDLMERLRPDAESNN   349
E. coil XerD         LLGHSDLSTTQIYTHVATERLRQLHQQHHPRA.......   298
Haemophilus phage    ILGHSTIEMTMRYAHFAPSHLESAVKFNPLSNPAQ....   337
S. warneri           YLGHQTTHETMKFIGIKDKMHNRTVIALNL.........   185
S. pasteuri          YLGHQTTHETMKFIGIKDKTHNNTVIALNL.........   185
S. capitis           YLGHQTTQETIKFIGIQDKSIHQTVIALNL.........   185
S. caprae            YLGHQTTQETMKFIGIKDKSIKRTVIALNL.........   185
S. haemolyticus      YLGHQTTQETVKFIGLSADDELHTIIALNL.........   185
S. hominis           YLGHQTTHETLKFIGLSPKEDHHTIIALNL.........   185
S. schweitzeri       YLGHQSTIETLNFIGVENESEHSIYITLNL.........   186
S. aureus            YLGHQSAIETLNFIGLENECEHSIYISLQL.........   186
S. lugdunensis       YLGHQTTQETMKFIGIKKIEKNQTVIALNL.........   185
```
