# Supplementary figures and images for: Identification of Three Clf-Sdr Subfamily Proteins in Staphylococcus warneri, and Comparative Genomics Analysis of a Locus Encoding CWA Proteins in Staphylococcus Species
Source: Front Microbiol. 2021 Jul 29;12:691087. doi: 10.3389/fmicb.2021.691087 (PMC8360574; doi:10.3389/fmicb.2021.691087)

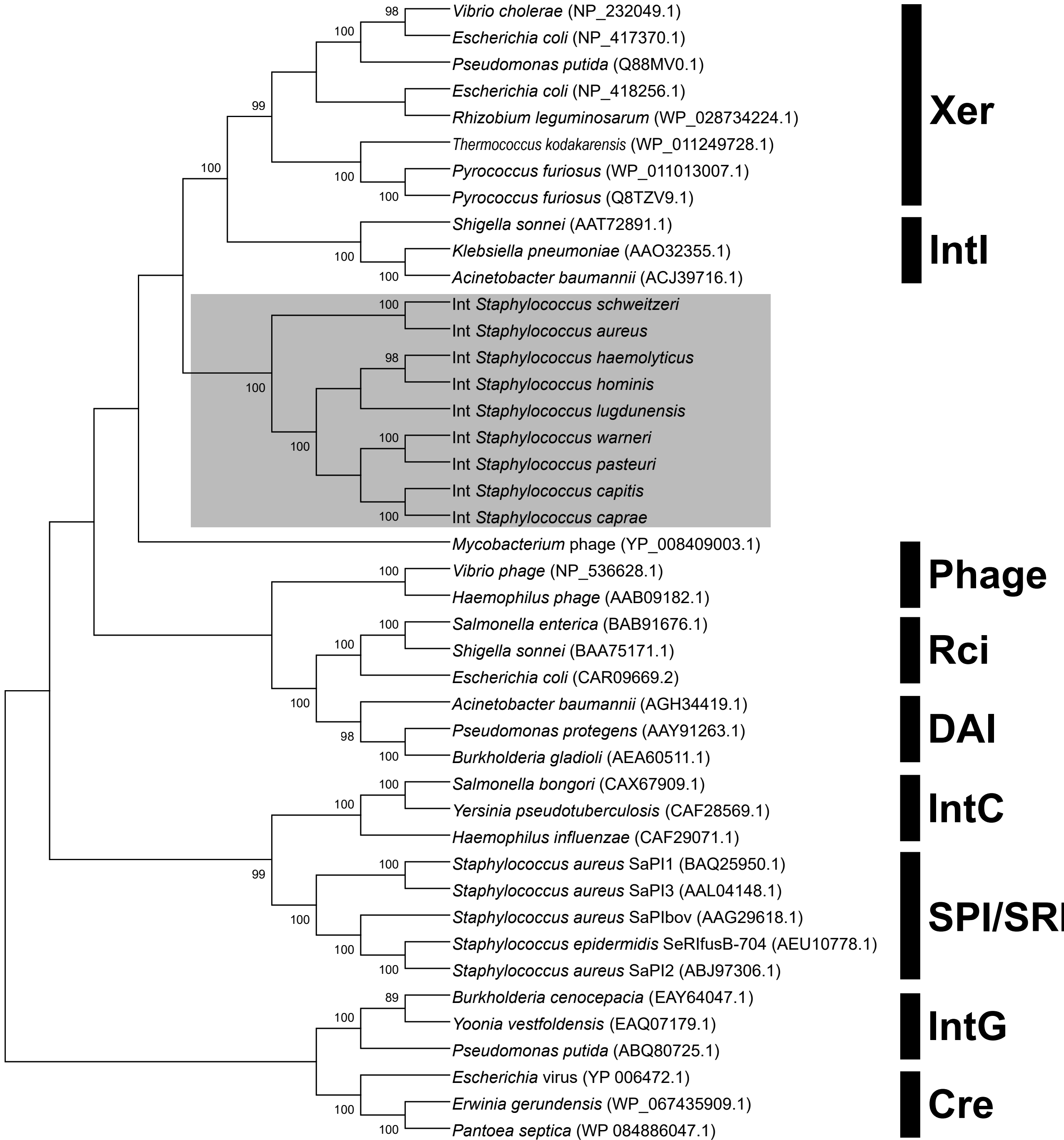

Supplement: Supplementary Figure 4 — The inferred phylogenetic relationship of the putative integerase on Sw-Sdr in S. warneri or Sw-Sdr related regions in other Staphylococcus species belong to group SD in relation to representative tyrosine recombinases of the shufflon-specific DNA recombinase (Rci), dusA-associated integrases (DAI), phage integrase (Phage), integrative and conjugative element (IntC), Staphylococcus pathogenicity island (SPI), Staphylococcus resistance island (SRI), integron integrase (IntI), site-specific recombinase (XerDC), phage P1-like recombinases (Cre) and genomic island integrase (IntG) families. Protein accession numbers of sequences used to generate this phylogenetic tree are in brackets. The interior values are the bootstrap probabilities after 1,000 replicates. [file Image_4.PDF]

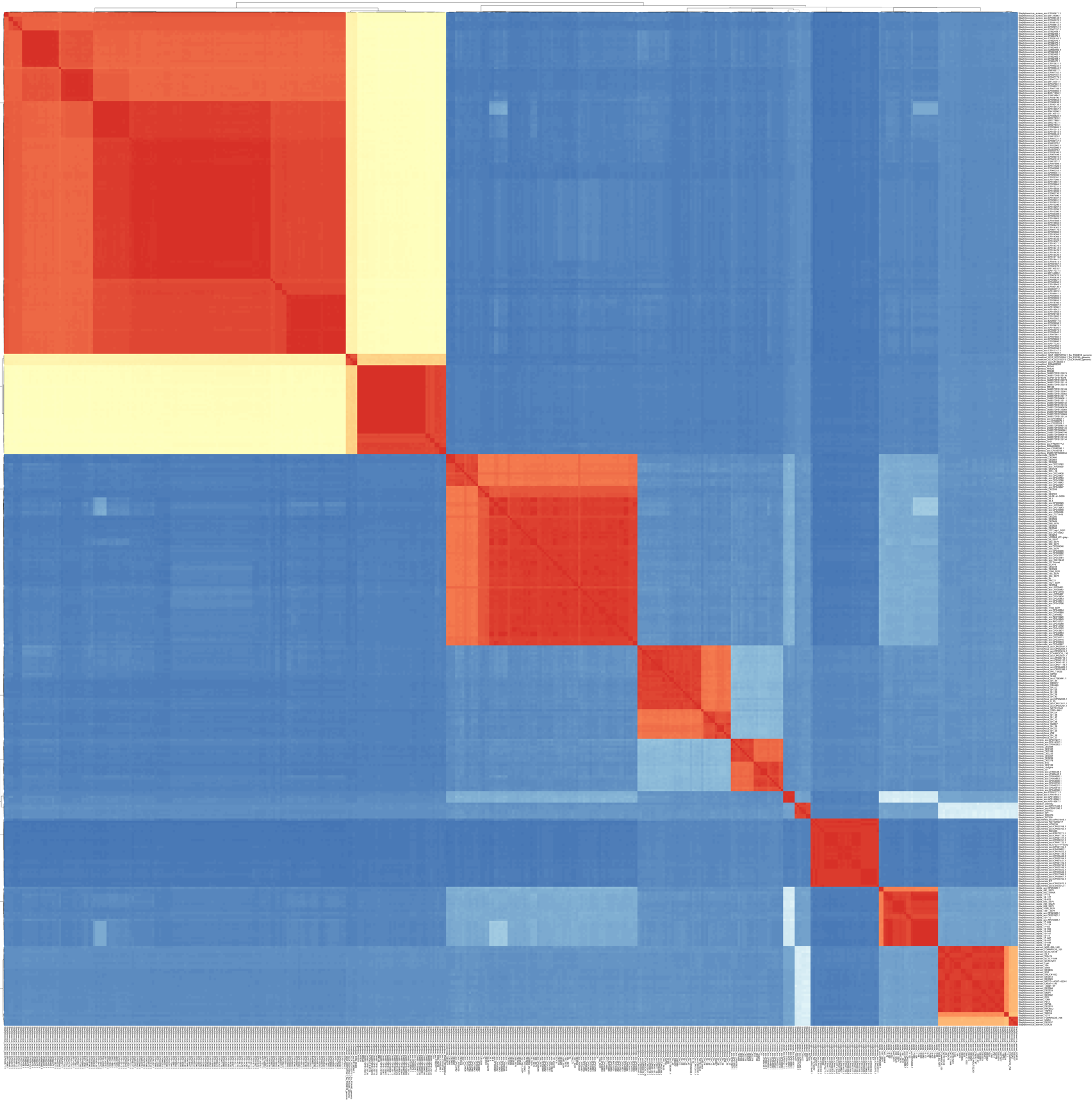

Supplement: Supplementary Figure 5 — Average nucleotide identity between each genome used to investigate the sdr locus. [file Image_5.PDF]
