# Supplementary material for: Identification of Three Clf-Sdr Subfamily Proteins in Staphylococcus warneri, and Comparative Genomics Analysis of a Locus Encoding CWA Proteins in Staphylococcus Species
Source: Front Microbiol. 2021 Jul 29;12:691087. doi: 10.3389/fmicb.2021.691087 (PMC8360574; doi:10.3389/fmicb.2021.691087)
Supplement: Supplementary Table 1 — Genome features of S. warneri WS479. [file Table_1.DOCX]

|  | **Chromosome** | **pWS-25** | **pWS-31** |
| --- | --- | --- | --- |
| **Size(bp)** | 2,508,234 | 25,160 | 31,000 |
| **GC content (%)** | 32.87 | 29.86 | 30.46 |
| **ORFs** | 2,491 | 33 | 34 |
| **Known proteins** | 2,135 | 6 | 26 |
| **Hypothetical protein** | 274 | 27 | 8 |
| **Protein coding (%)** | 96.71 | 100.00 | 100.00 |
| **Average ORF length (bp)** | 878 | 605 | 703 |
| **Average protein length (aa)** | 297 | 201 | 233 |
| **tRNAs** | 62 | 0 | 0 |
| **rRNAs** | (16S-23S-5S) * 6 (16S-23S-5S-5S) * 0 | 0 0 | 0 0 |

Table S1

Genome features of *S. warneri* WS479
