# Supplementary material for: Identification of Three Clf-Sdr Subfamily Proteins in Staphylococcus warneri, and Comparative Genomics Analysis of a Locus Encoding CWA Proteins in Staphylococcus Species
Source: Front Microbiol. 2021 Jul 29;12:691087. doi: 10.3389/fmicb.2021.691087 (PMC8360574; doi:10.3389/fmicb.2021.691087)
Supplement: Supplementary Table 2 — MIC values of antibacterial drugs for all strains (μg/mL). [file Table_2.DOCX]

**Table S2**

MIC values of antibacterial drugs for all strains (µg/mL).

|  | *S.warneri* WS479 | ATCC 29212 | ATCC 25922 | JH2-2 | pAM401/JH2-2 | pAM401-*aadD2*/JH2-2 | DH5α | pUCP24/DH5α | pUCP24-*blaZ*/DH5α |
| --- | --- | --- | --- | --- | --- | --- | --- | --- | --- |
| TOB | 4 | 8 | - | 32 | 32 | 256 | - | - | - |
| AMK | 256 | 256 | - | 256 | 256 | >512 | - | - | - |
| GEN | <0.25 | 4 | 0.5 | 8 | 8 | 8 | - | - | - |
| KAN | 4 | 32 | 4 | 64 | 64 | >1024 | - | - | - |
| STR | <1 | 64 | 4 | 128 | 128 | 128 | - | - | - |
| AMP | 2 | 2 | 4 | - | - | - | 4 | 2 | >1024 |
| FOX | 4 | 256 | 2 | - | - | - | 2 | 2 | 2 |
| CZO | <1 | 16 | 2 | - | - | - | <1 | 2 | 256 |
| CAZ | 16 | 512 | 0.25 | - | - | - | 0.25 | <0.06 | 16 |
| CTX | 2 | 128 | 0.06 | - | - | - | <0.06 | <0.06 | <0.06 |
| MEM | 0.06 | 4 | 0.06 | - | - | - | <0.03 | <0.03 | >32 |
| CIP | 0.03 | 2 | 0.03 | - | - | - | - | - | - |
| PEN | <1 | 4 | - | - | - | - | - | - | - |
| AZM | 128 | 2 | - | - | - | - | - | - | - |
| ERY | 64 | 1 | 32 | - | - | - | - | - | - |
| ROX | 256 | 1 | - | - | - | - | - | - | - |
| NAL | 128 | 1024 | <1 | - | - | - | - | - | - |
| CLR | 32 | <1 | 32 | - | - | - | - | - | - |

TOB, Tobramycin; GEN, Gentamicin; KAN, Kanamycin; STR, Streptomycin; AMP, Ampicillin; FOX, Cefoxitin; CZO, Cephazolin; CAZ, Ceftazidime; CTX, Cefotaxime; MEM, Meropenem; CIP, Ciproﬂoxacin; PEN, Penicillin; AMK, Amikacin; AZM, Azithromycin; ERY, Erythromycin; ROX, Roxithromycin; NAL, Nalidixic acid; CLR, Clarithromycin. ATCC 29212, *Enterococcus faecalis* used as the quality control strain for the antimicrobial test; ATCC 25922, *Escherichia coli* used as the quality control for antimicrobial susceptibility testing; JH2-2, *Enterococcus faecalis* used as the host for cloned *aadD2* gene; DH5α, *Escherichia coli* used as a host for the cloned *blaZ* gene.
