# Supplementary material for: Identification of Three Clf-Sdr Subfamily Proteins in Staphylococcus warneri, and Comparative Genomics Analysis of a Locus Encoding CWA Proteins in Staphylococcus Species
Source: Front Microbiol. 2021 Jul 29;12:691087. doi: 10.3389/fmicb.2021.691087 (PMC8360574; doi:10.3389/fmicb.2021.691087)

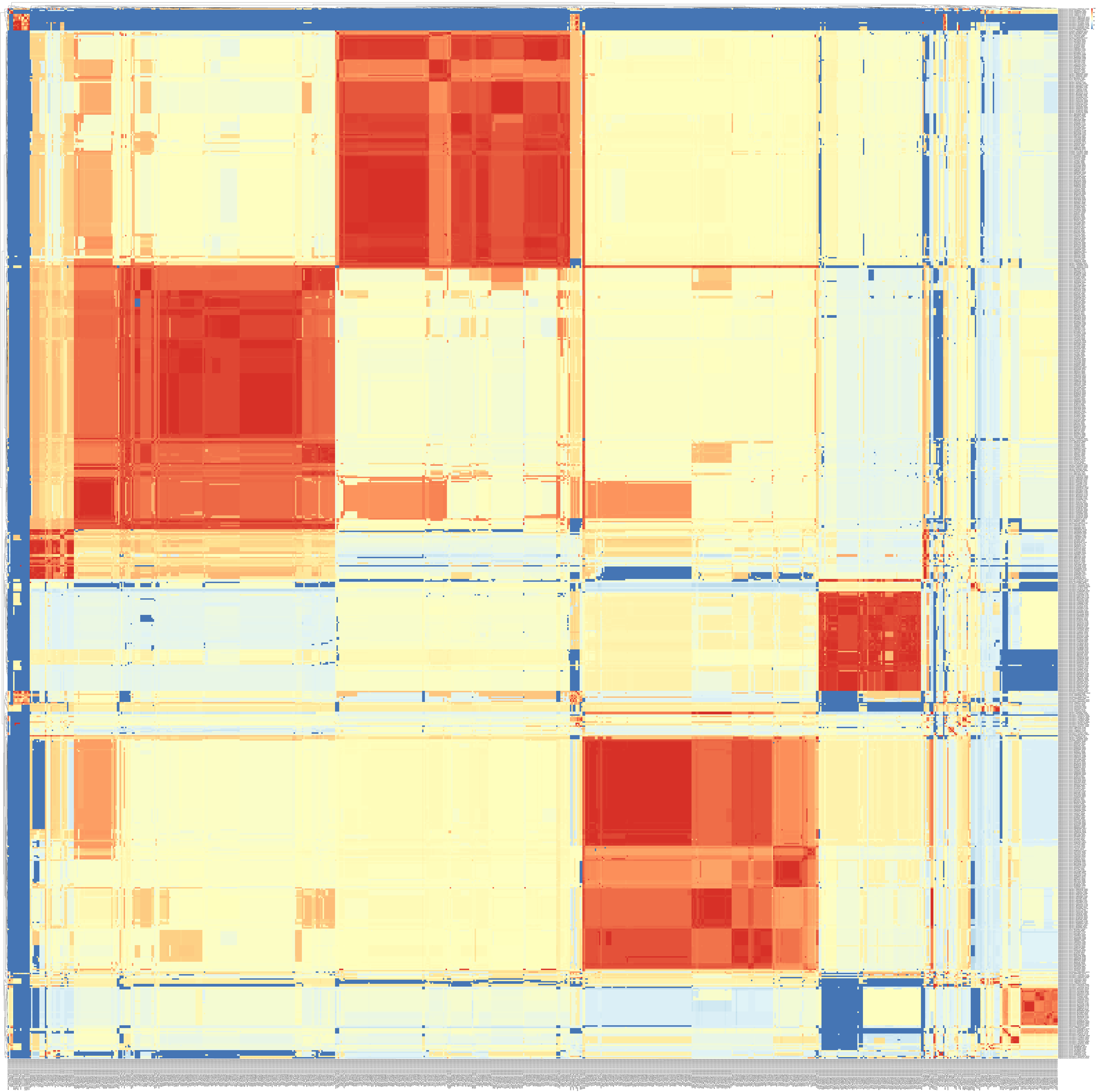

Supplement: Supplementary Figure 6 — Sequence identity comparison of CWA proteins (identified based on the presence of key domains) encoded at sdr loci in Staphylococcus species belonging to group SD. [file Image_6.PDF]
